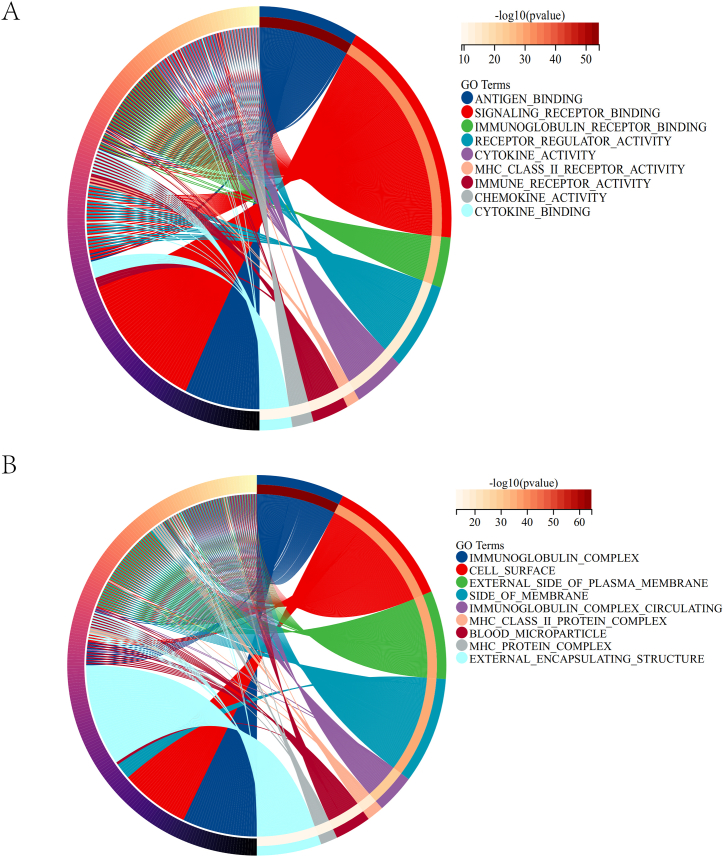# Cervical cancer subtype identification and model building based on lipid metabolism and post-infection microenvironment immune landscape

**DOI:** 10.1016/j.heliyon.2024.e30746

**Published:** 2024-05-04

**Authors:** Yongzhi Chen, Rongjie Cui, Dun Xiong, Yuan Zhao, Jianyu Pang, Samina Gul, Qi Qi, Yuheng Tang, Xuhong Zhou, Wenru Tang

**Affiliations:** a Medicine School, Kunming University of Science and Technology, Kunming, PR China; b Department of Thyroid and Breast Surgery, Pu'er People's Hospital, Puer, Yunnan, PR China; c Office of Science and Technology, Yunnan University of Chinese Medicine, Kunming, PR China

**Keywords:** scRNA-seq, PIM, LMRGs, Targeted therapy, Molecular docking

## Abstract

**Background:**

As the second most common gynecological cancer, cervical cancer (CC) seriously threatens women's health. The poor prognosis of CC is closely related to the post-infection microenvironment (PIM). This study investigated how lipid metabolism-related genes (LMRGs) affect CC PIM and their role in diagnosing CC.

**Methods:**

We analyzed lipid metabolism scores in the CC single-cell landscape by AUCell. The differentiation trajectory of epithelial cells to cancer cells was revealed using LMRGs and Monocle2. Consensus clustering was used to identify novel subgroups using the LMRGs. Multiple immune assessment methods were used to evaluate the immune landscape of the subgroups. Prognostic genes were determined by the LASSO and multivariate Cox regression analysis. Finally, we perform molecular docking of prognostic genes to explore potential therapeutic agents.

**Results:**

We revealed the differentiation trajectory of epithelial cells to cancer cells in CC by LMRGs. The higher LMRGs expression cluster had higher survival rates and immune infiltration expression. Functional enrichment showed that two clusters were mainly involved in immune response regulation. A novel LMR signature (LMR.sig) was constructed to predict clinical outcomes in CC. The expression of prognostic genes was correlated with the PIM immune landscape. Small molecular compounds with the best binding effect to prognostic genes were obtained by molecular docking, which may be used as new targeted therapeutic drugs.

**Conclusion:**

We found that the subtype with better prognosis could regulate the expression of some critical genes through more frequent lipid metabolic reprogramming, thus affecting the maturation and migration of dendritic cells (DCs) and the expression of M1 macrophages, reshaping the immunosuppressive environment of PIM in CC patients. LMRGs are closely related to the PIM immune landscape and can accurately predict tumor prognosis. These results further our understanding of the underlying mechanisms of LMRGs in CC.

## 1 Introduction

CC is a cancer with high mortality caused by human papillomavirus. In women, CC is a major cause of death [1]. Conventional cancer treatment can improve clinical outcomes, but the prognosis of CC is still poor [2].

Immunotherapy is a novel approach to cancer treatment that may help improve the potential impact of increased immunotherapy by developing novel biomarkers related to tumor immune response [3]. We can establish a risk stratification approach and screen prognostic genes to provide evidence for targeted therapy of CC.

The vast majority of CCs are associated with HPV infection. The main reason for the virus's continuous propagation and malignant progression is that the cells infected with HPV change the local microenvironment [4]. It is essential to understand the immune landscape of PIM in order to treat CC.

HPV mainly infects keratinocytes [5]. Keratinocytes have a variety of pathogen recognition receptors that recognize invading pathogens and activate immune cells. HPV-regulated innate immune receptors and components of antigen processing and presentation mechanisms in infected keratinocytes play essential roles in helping the immune system recognize and promote the formation of PIM. The weakened immune response in HPV-infected keratinocytes affects the alarm system of surrounding immune cells, forming an immunosuppressive microenvironment [6].

LMRGs reprogramming has been proposed as a novel marker of tumor malignancy, and its role in tumor development and treatment has been demonstrated [7]. HPV causes the cells in the PIM to undergo metabolic reprogramming. Evidence shows that disruption of normal cell metabolism may result from an underlying HPV-mediated ecological imbalance [8]. Targeted lipid metabolism has been proposed as a new cancer therapy, but there is little research on CC.

It has been suggested that the immune landscape of CC involves a variety of immune cells and signaling pathways, and lipid metabolism may play an essential role in this process. Firstly, lipid metabolism can affect the lipid composition of cell membranes, affecting receptor expression, signaling, and function of immune cells. Secondly, lipid metabolism can regulate immune cell activity and function. For example, lipid metabolites such as lipid analogs and liposomes can act as signaling molecules to regulate immune cell polarization, secretion of immune factors, antigen presentation, and cell killing. Thirdly, lipid metabolism regulates various signaling pathways, such as mTOR, PPAR, NF-κB, and other signaling pathways, which play an essential role in regulating the activity and function of immune cells and modulating immune-inflammatory responses. Finally, abnormal lipid metabolism may lead to immune tolerance and escape from tumor cells [9].

This research focuses on the influence of LMRGs on the PIM of CC patients. A novel six LMRGs signature (*SLC10A2*, *THRSP*, *PTGIS*, *SLC25A17*, *PLAAT2*, and *PIP4K2A*) was constructed to predict clinical outcomes in CC, which are listed in Table 1. We developed an LMR.sig to assess the predictive significance of LMRGs in CC. The importance of prognostic genes was verified from various perspectives, and potential targeted drugs were screened. Our study sheds new light on the personalized treatment of CC.

**Table 1:** | The genes used for constructing the LMR.sig.

Gene name	Full name	Function	Relationship to prognosis
*SLC10A2*	Solute Carrier Family 10 Member 2	This gene encodes a sodium/bile acid cotransporter.	HR > 1:Associated with poor prognosis
*THRSP*	Thyroid Hormone Responsive	The protein encoded by this gene is limited to the liver and adipose tissue and is controlled by nutritional and hormonal factors.	HR > 1:Associated with poor prognosis
*PTGIS*	Prostaglandin I2 Synthase	This gene encodes a member of the cytochrome P450 superfamily of enzymes.	HR > 1:Associated with poor prognosis
*SLC25A17*	Solute Carrier Family 25 Member 17	This gene encodes a peroxisomal membrane protein belonging to the mitochondrial solute carrier family.	HR < 1:Associated with good prognosis
*PLAAT2 (HRASLS2)*	Phospholipase A And Acyltransferase 2	The protein encoded by this gene has phospholipase and acyltransferase activities and is a tumor suppressor.	HR < 1:Associated with good prognosis
*PIP4K2A*	Phosphatidylinositol-5-Phosphate 4-Kinase Type 2 Alpha	This gene regulates secretion, cell proliferation, differentiation, and motility.	HR < 1:Associated with good prognosis

## 2 Materials and methods

### 2.1 Data sources and pre-processing

We obtained a single-cell CC (GSE168652) dataset from the GEO database (Accessed May 10, 2022), including 2 case samples. RNA-seq, mutation, and related clinical information of CC patients were collected from UCSC databases (Accessed May 25, 2022). RNA-seq data were included from 309 samples, including 306 CC and three normal samples. RNA-seq data were normalized by log2 (x+1). After excluding the samples without patient survival time or survival status in clinical information, the RNA expression data of 306 patients with CC were randomly allocated to a 3/4 training set (n = 229) and 1/4 internal validation set (n = 77). Finally, download a data set from the GEO database (GSE30760) and select the CC samples as the external verification set (n = 63). Datasets of 743 LMRGs were obtained from the Molecular Signatures.

### 2.2 Single-cell cell type annotations and epithelial cell/cancer cell distinction

We used the R package Seurat to analyze the single cell data from CC and got 24498 cells. We performed quality control according to the criteria nFeature_RNA >500 & nFeature_RNA <5000 & percent.mt < 10 & nCount_RNA >200 & nCount_RNA <35000, and finally obtained 11394 cells. The remaining cells were normalized and linearly downscaled by principal component analysis. Cells were clustered using the KNN algorithm, and UMP selected appropriate cell sub-clusters. We labeled the cell subclusters using the top 10 marker genes and the Human Protein Atlas (HPA) database. We evaluated the correlation between LMRGs.Score and annotate cells using the AUCell algorithm. The basic principle of the AUCell algorithm is to utilize gene expression data and a given set of genes to assess the relative abundance of each cell.

We used the copyKAT algorithm to distinguish Epithelial cells/Cancer cells. copyKAT algorithm can differentiate between normal and malignant cells in TME; it distinguishes between aneuploid and aneuploid cells by calculating the DNA copy number events. Aneuploid cells are considered tumor cells, whereas normal stromal cells and immune cells usually have a 2 N diploid or near diploid copy number. We then evaluated the correlation of LMRGs.Score with epithelial cells and cancer cells by AUCell.

### 2.3 Pseudo-time and somatic mutation analysis

The Monocle algorithm arranges cells into trajectories with branching points based on a specific set of genes input through a machine learning approach. We constructed the proposed temporal differentiation trajectories of epithelial and cancer cells by LMRGs and Monocle2. CC somatic mutation analysis was then performed using the R package maftools.

### 2.4 Identiﬁcation of molecular subtypes

ConsensusClusterPlus [10] was employed to conduct cluster analysis based on the expression matrix of LMRGs. Subsequently, we analyzed the optimal cluster, and this procedure was performed 1000 times to ensure the stability of the results.

### 2.5 Immune landscape evaluation

IOBR [11] is an immune tumor biology computing tool. Here, we select ESTIMATE and TIMER. The ESTIMATE method was employed to compute the stromal, immune, and ESTIMATE scores. The TIMER algorithm calculated the relative percentage of six immune cells.

### 2.6 Functional analyses

Limma [12] is a differential expression screening method based on generalized linear models. We used the R package limma for differential analysis to extract the differential genes (DEGs). To enrich related pathways, we performed to determine the diﬀerence between clusters using Gene Ontology (GO), Kyoto Encyclopedia of Genes and Genomes (KEGG), and Gene Set Enrichment Analysis (GSEA).

### 2.7 Development and verification of LMR.sig

Regression analysis using the Lasso-Cox approach and tenfold cross-validation was performed to select the best model. Multivariate COX regression analysis was then used to identify the genes needed to develop the LMR.sig. Using the R package pROC for ROC analysis, the AUC was calculated. ROC analyses were conducted at 365-day, 1095-day, and 1825-day periods using pROC's ROC function, and AUC and confidence intervals were determined using pROC's CI function to generate the final AUC values. Then, we used the “rms” R package to build a nomogram using the Cox method and analyze the predictive significance of these dates.

We downloaded the simple nucleotide variation dataset of CC samples processed by MuTect2 software [13] from Genomic Data Commons (GDC, Accessed Oct 5, 2022). We used R package maftools of the tmb function to calculate the tumor mutation burden (TMB) of CC.

### 2.8 Prognostic genes expression and immune landscape

We obtained immunohistochemical data of prognostic genes from the HPA database (Accessed Oct 15, 2022) by R package HPAanalyze for evaluating the expression of prognostic genes in CC and normal tissues.

We used the UCSC database to download a standardized universal cancer dataset. We transformed each expression value using the log2 (x+0.001) transformation.

The infiltration score of 22 types of immune cells for each patient was evaluated using IOBR's CIBERSORT method based on gene expression. We used the corr. Test function of the R software package “Psych” to calculate the correlation coefficient between prognostic genes and immune cell scores in each tumor sample.

### 2.9 Drug screened and docking

We first found the CTD numbers of compounds interacting with essential genes through the Comparative Toxicogenomics Database (CTD, Accessed Dec 16, 2022) database, of which 136 compounds were interacting with *SLC10A2*, 141 compounds interacting with *THRSP*, 106 compounds interacting with *PTGIS*, 33 compounds interacting with *SLC25A17*, 30 compounds interacting with *HRASLS2*, and *116* compounds interacting with *PIP4K2A*. Then we obtained the pdb protein structures of the corresponding proteins of *PTGIS* (3b6h.pdb), *HRASLS2* (4dpz.pdb), and *PIP4K2A* (6ym5.pdb) obtained by X-ray method through Protein Data Bank (PDB, Accessed Dec 20, 2022) database. Protein structures obtained without the X-ray method were obtained by AlphaFold Protein Structure (Accessed Dec 20, 2022) Database by artificial intelligence for *SLC10A2* (AF-Q12908-F1-model_v4.pdb), *THRSP* (AF-Q92748-F1-model_v4.pdb) and *SLC25A17* (AF-O43808-F1-model_v4.pdb) pdb protein structures. Then, we obtained the sdf files of the corresponding compounds according to the compound CID numbers through shell scripts and converted each sdf file to mol2 format using OpenBabel. Then, we use Autodock (Linux, v4.2) to dock the receptor and ligand molecularly and finally get the result file. The unit of binding energy in the result is kcal/mol; the smaller the binding energy is, the easier it is for the small molecule receptor and ligand to bind. Then we transformed the pdb results of the proteins corresponding to the critical genes into pdbqt structures by OpenBabel, and then combined the pdbqt and the best dlg result files respectively by Autodock, and finally visualized the results by Pymol (v2.6).

### 2.10 Statistical analyses

Survival studies were conducted using Kaplan-Meier (KM) analysis. A non-parametric test was used for the inter-group test, and Spearman rank correlation analysis was used to calculate the correlation. p < 0.05 or FDR <0.05 were considered statistically significant. Statistical analyses were conducted via R (v4.1.3).

Fig. S1 shows the entire data analysis procedure.

## 3 Results

### 3.1 LMRGs.Score in single-cell landscapes and epithelial/cancer cells

All cells were descendingly clustered into ten subclusters (Fig. 1A). These ten subclusters were further annotated into six cell types: Epithelial cells/Cancer cells, Smooth muscle cells, Endothelial cells, Fibroblasts, NK cells, and Macrophages (Fig. 1B). Bubble plots demonstrate the expression levels of marker genes in these six cell types (Fig. 1C).

**Fig. 1:**
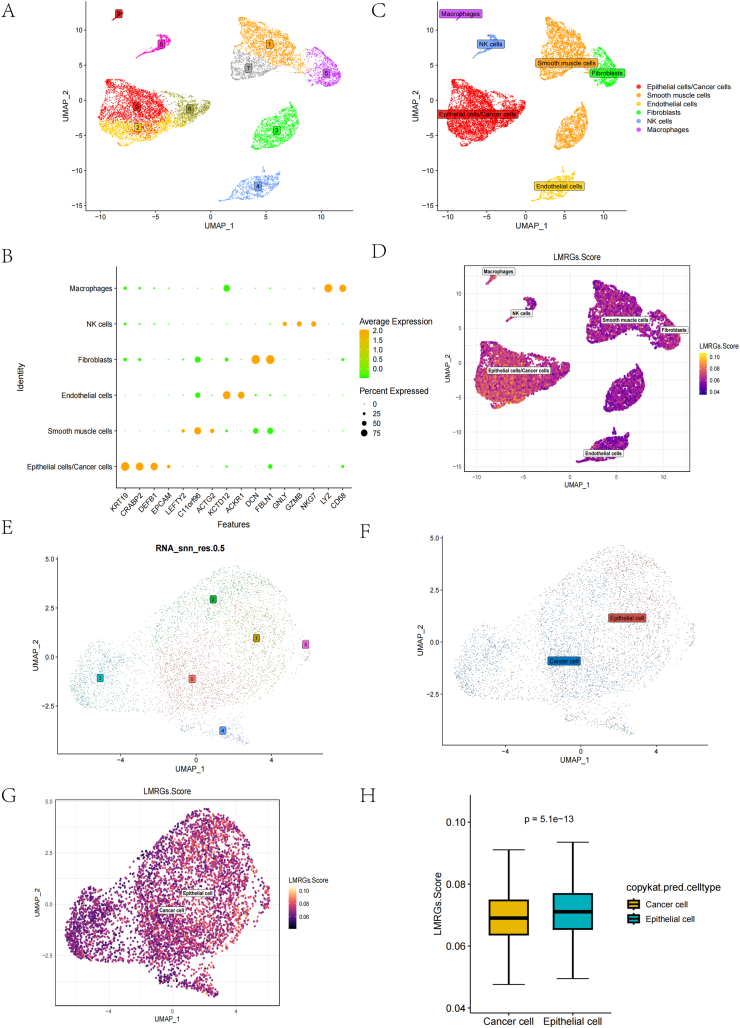
Cell type annotation and assessment of lipid metabolism scores. (A) Ten cell clusters were obtained after downscaling. (B) Bubble plots show marker genes' expression levels in the six cell types. (C) Six cell types were identified by marker gene annotation. (D) Lipid metabolism scores in the CC single-cell landscape. (E) Ten cell clusters were obtained after downscaling. (F) The copyKAT algorithm distinguishes epithelial cells from cancer cells. (G, H) The LMRGs.Score correlation between epithelial cells and cancer cells.

We analyzed the LMRGs.Score in the CC single-cell landscape by AUCell and found that the LMRGs.Score had the highest expression in Epithelial cells/Caner cells (Fig. 1D), and there were significant differences between these cell types (Fig. S2A, ****p < 0.0001). These findings suggest that lipid metabolism in CC may be closely related to Epithelial cells/Cancer cells in PIM.

We presented Epithelial cells/Cancer cells individually, and after reclustering by dimensionality reduction, all cells of Epithelial cells/Cancer cells were dimensionalized into six cell clusters (Fig. 1E). We distinguished epithelial and cancer cells from these six cell clusters using the copyKAT algorithm (Fig. 1F). We then assessed the LMRGs.Score between epithelial cells and cancer cells using AUCell, and we found that the LMRGs.Score in epithelial cells was significantly higher than that in cancer cells (p = 5.1e-13, Fig. 1G and H).

### 3.2 Construction of differentiation trajectories of epithelial cells and cancer cells by LMRGs

We constructed the differentiation trajectory from epithelial and cancer cells using LMRGs and Monocle (Fig. 2A). Studies have shown that cancer cells are de-differentiated compared to epithelial cells. We found that cancer cells are in the early stages, and epithelial cells are in the middle to late stages of differentiation (Fig. 2B). The results show that our lipid metabolism may be related to tumor Epithelial Mesenchymal Transition (EMT).

**Fig. 2:**
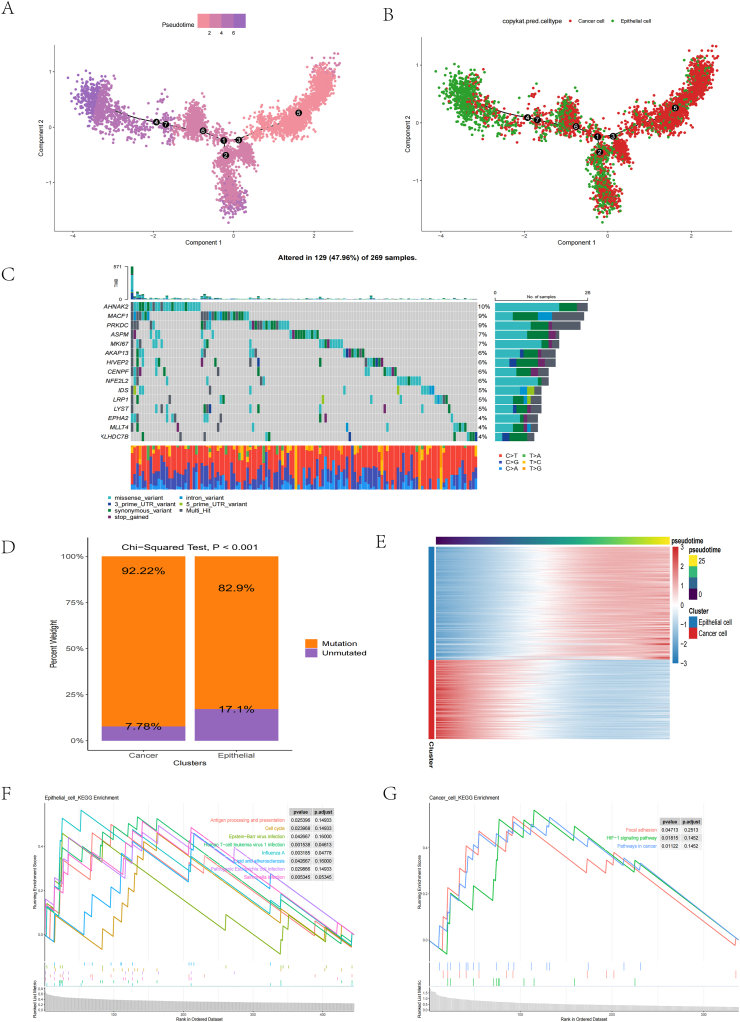
Differentiation trajectory construction and mutations landscape of epithelial cells and cancer cells. (A) Monocle constructed pseudotime for epithelial cells and cancer cells. (B) The differentiation trajectory of epithelial cells to cancer cells was constructed from LMRGs. (C) Waterfall plot visualizing the top 15 genes in terms of mutation frequency. (D) Mutation status of differentiated genes in epithelial and cancer cells. Based on the differentially differentiated genes (E), KEGG analysis was performed to analyze the significantly enriched pathways in epithelial (F) and cancer cells (G).

To further demonstrate that the differentiation trajectory from epithelial to cancer cells in CC is related to tumor EMT, we visualized the expression of some classical EMT-related genes (*TGFB1*, *CDH1*, *STAT3,* and *HIF1A*) in epithelial and cancer cell differentiation trajectories, and found that the four classical EMT-related genes were all highly expressed in epithelial, and were lowly expressed in cancer cells (Figs. S2B–E). *TGFB1* is a multifunctional cytokine that plays a crucial role in EMT by regulating cell adhesion, basement membrane breakdown, and junctions between epithelial cells. *TGFB1* can induce the transformation of epithelial cells into mesenchymal cells. It promotes transcriptional changes between cells, leading to epithelial cells gradually losing their epithelial properties and becoming more like mesenchymal cells [14]. *CDH1* is an essential intercellular adhesion protein maintaining epithelial cell polarity and structure. CDH1 can weaken adhesion between epithelial cells during EMT, promoting cell separation and movement [15]. *STAT3* is a transcription factor involved in cell signaling and gene regulation in various biological processes. In EMT, *STAT3* has been found to regulate the expression of transcription factors, such as Snail and Twist, which inhibit the expression of epithelial cell markers and promote EMT [16]. *HIF1A* is a critical transcription factor in response to hypoxic environment. Hypoxia is expected in the tumor microenvironment and has been shown to play an essential role in regulating EMT in tumor cells. *HIF1A* can promote EMT through various pathways, including controlling the expression of epithelial-mesenchymal transcription factors and enhancing cell invasiveness and migration [17]. This provides more detailed evidence that the differentiation trajectory of epithelial cells to cancer cells in CC is associated with tumor EMT.

We visualized the top 15 mutated genes among differentiated genes in epithelial cells and cancer cells, and the mutation rate of *AHNAK2* reached 10 % (Fig. 2C). The results showed that the mutation rate of differentiated genes in cancer cells was 92.22 %. In comparison, that of epithelial cells was 82.9 %, which is consistent with our knowledge that cancer cells are characterized by high mutability (Fig. 2D). We performed KEGG enrichment analysis based on the differentiation genes associated with cancer cells and epithelial cells (Fig. 2E). The results showed that antigen processing, presentation, and cell cycle pathways were significantly upregulated in epithelial cells (Fig. 2F); cancer-related pathways were significantly upregulated in cancer cells (Fig. 2G).

### 3.3 Consensus clustering identiﬁcation of two LMRGs molecular clusters

We carried out the univariate COX regression analysis of 745 LMRGs and found 85 genes associated with survival (p < 0.05). The forest plot reveals 85 LMRGs linked to CC prognosis (Fig. S3). Using RNA-seq expression data of 85 genes, we divided patients with CC into subgroups via consensus clustering. Using the empirical cumulative distribution function plot, we found the optimal clustering stability when K = 2 (Fig. 3A–C). The survival analysis of the two clusters showed a significant difference between C1 and C2 (p = 6.4e-6, Fig. 3D). In addition, a heatmap showed the level of LMRGs expression in the two subtypes, clusters C1 and C2, exhibit significant expression differences (Fig. 3E).

**Fig. 3:**
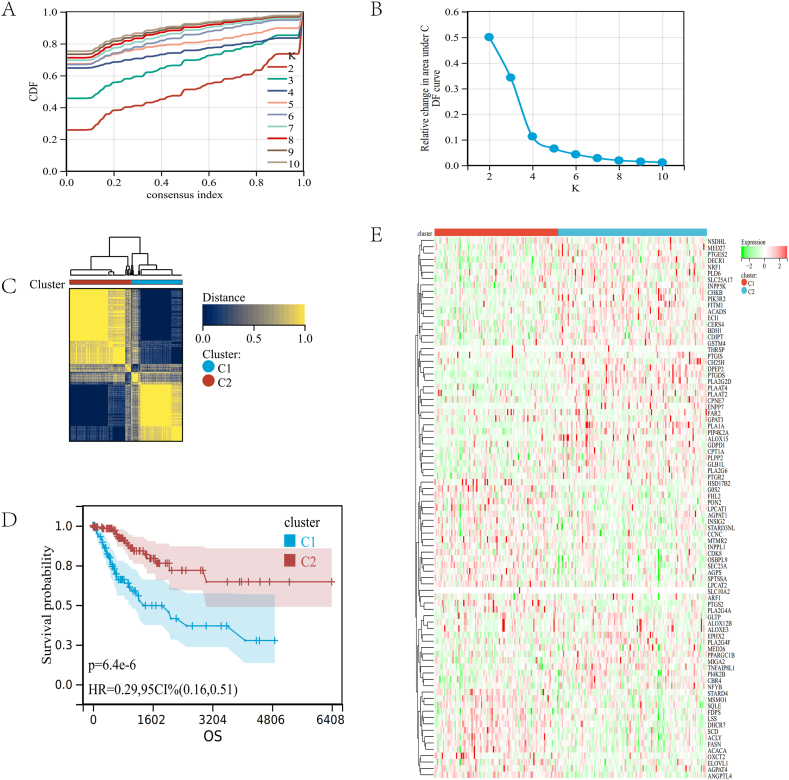
Consensus clustering. (A–C) The consensus cluster, K = 2, was determined to be the optimal value. (D) Survival curves for the two subtypes of patients. (E) The heatmap in the two subtypes.

These findings indicated that the LMRGs divide individuals with CC into two distinct molecular subgroups, and the C2 cluster has a high expression of LMRGs and a higher survival rate.

### 3.4 Two molecular subgroups displayed distinct immune landscapes of PIM

**Fig. 4:**
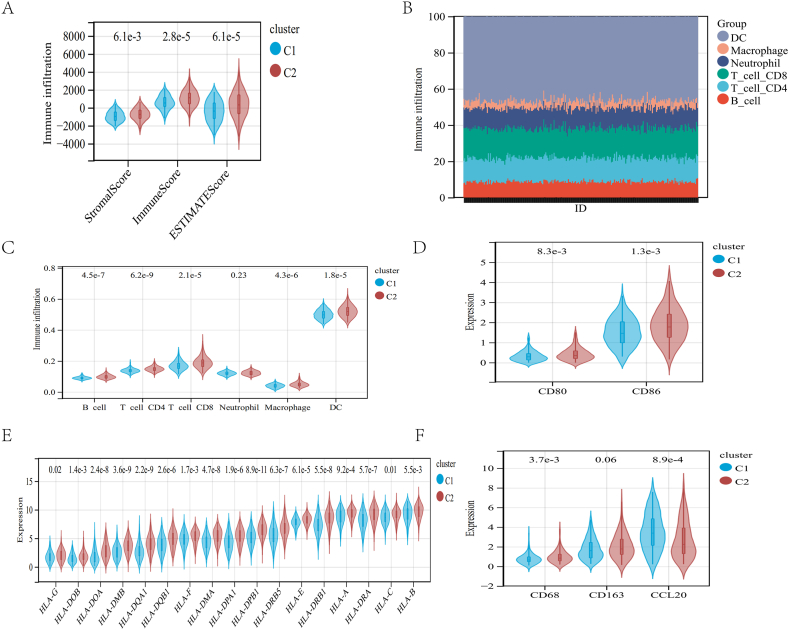
Immune landscape analysis. (A) The ESTIMATE algorithm evaluated the immune microenvironment. (B, C) The relative proportion of immune infiltrating cells was evaluated by the TIMER algorithm. (D) Evaluation of *CD80* and *CD86* expression. HLA genes (E) were evaluated between the two groups. (F) Evaluation of *CD68, CD163,* and *CCL20* expression.

Then, we identified the immune differences between the two molecular subgroups. Cluster C2 patients with CC had significantly higher stromal scores (P = 6.1e-3), immune scores (P = 2.8e-5), and ESTIMATE scores (P = 6.1e-5) via ESTIMATE analysis (Fig. 4A). The C2 cluster had a higher survival rate and a higher immune status.

We employed the TIMER approach to analyze differences between the two subtypes in the immune infiltration of six immune cells (Fig. 4B). And in cluster C2, TIMER analysis showed that CC patients had significantly higher B cells (P = 4.5e-7), CD4 T cells (P = 6.2e-9), CD8 T cells (P = 2.1e-5), macrophages (P = 4.3e-6), and DCs (P = 1.8e-5) compared with cluster C1 (Fig. 4C).

Keratinocytes secretion of immunosuppressive regulatory factors such as *IL-6*, *IL-10*, and *PGE2* limits DCs maturation and is characterized by reduced expression of cell surface co-stimulatory molecules such as major histocompatibility complex (MHC), *CD80*, and *CD86* [18,19]. Therefore, the expressions of *CD80* and *CD86* in the two clusters were also evaluated, and it was found that the expressions of *CD80* (P = 8.3e-3) and *CD86* (P = 1.3e-3) in the C2 cluster were significantly higher than those of C1 cluster (Fig. 3D). Human leukocyte antigen (HLA, MHC) is a class of protein molecules responsible for antigen presentation on the surface of antigen-presenting cells. HLA family genes were also evaluated separately. Similarly, the expression of these genes in the C2 cluster was significantly higher than that in the C1 cluster (Fig. 4E).

Abundant evidence suggests that *CD68* is a marker of M1 macrophages and *CD163* is a marker of M2 macrophages [20]. We found that *CD68* and *CD163* were higher in C2 than in C1, and the expression of *CD68* (P = 3.7e-3) was significantly higher. (Fig. 4F).

*CCL20* can induce the accumulation of immature DCs so that the body cannot initiate the immune response against the tumor [21]. We found that the expressions of *CCL20* (P = 8.9e-4) in the C1 cluster were significantly higher than those in the C2 cluster (Fig. 4F).

These findings suggest that the two molecular clusters can form significantly different immune patterns by influencing the maturation and metastasis of immune cells and that the expression of LMRGs is related to the immune pattern.

### 3.5 Difference and functional analyses in CC

**Fig. 5:**
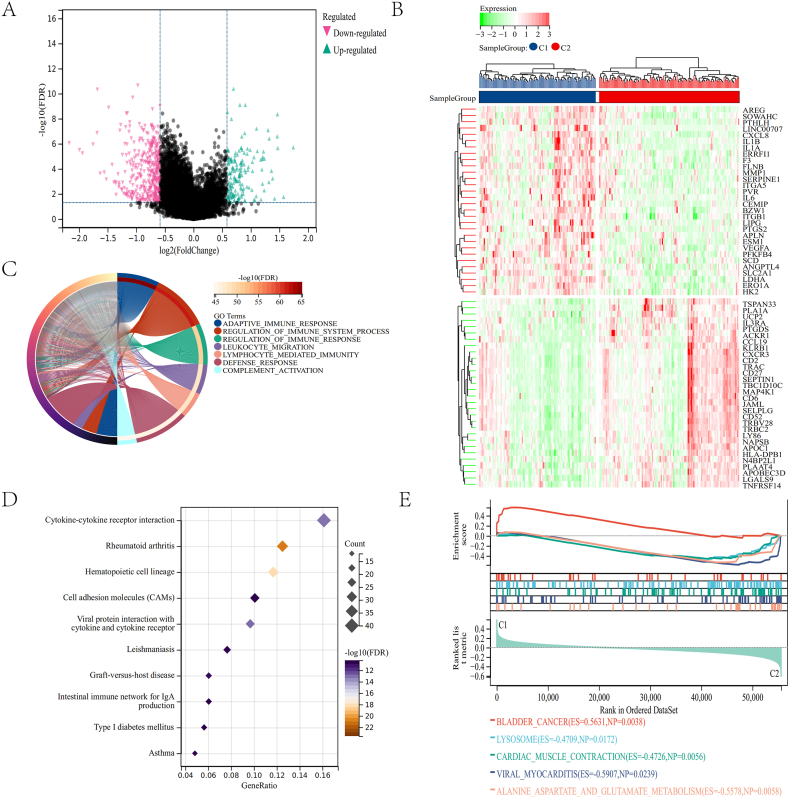
Functional enrichment analysis. (A, B) The volcano plot and heatmap show the DEGs between the two subtypes. (C) Circle plot illustrating the biological processes enriched by GO analysis. (D) Bubble diagram illustrating the signaling pathways enriched by KEGG analysis. (E) Plots visualize the results enriched by GSEA.

We found DEGs between the two clusters and conducted functional enrichment studies to investigate the underlying signaling pathways. The significance threshold was FDR <0.05, and the difference between the two groups was 1.5 times. Compared to clusters C1 and C2, 534 DEGs were identified, 371 genes were down-regulated, and 163 were up-regulated (Fig. 5A). The expression level of LMRGs between clusters C1 and C2 was visualized through the heatmap (Fig. 5B).

The GO enrichment analysis revealed that the DEGs between clusters C1 and C2 were enriched in adaptive immune response and response regulation (Fig. 5C). Essential molecular functions and cellular components were enriched (Figs. S4A and B).

KEGG analysis found several critical pathways linked with rheumatoid arthritis and cytokine-cytokine receptor interaction between clusters C1 and C2 (Fig. 5D).

GSEA analysis revealed that the pathways in the two clusters were mainly enriched to alanine aspartate and glutamate metabolism, further indicating their relationship with the prognosis of CC (Fig. 5E).

These results suggest a correlation between LMRGs expression and activation and regulation of immune responses.

### 3.6 Development and validation of a LMR.sig

**Fig. 6:**
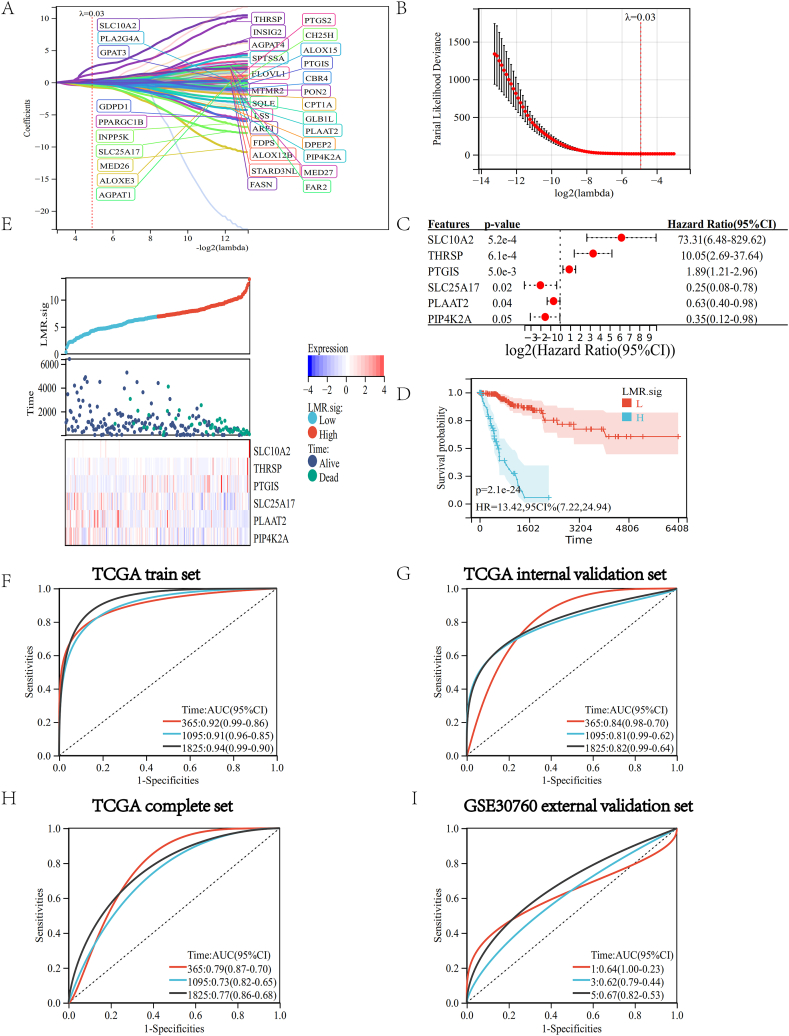
Construct and validate the CC model. (A, B) The LASSO analysis. (C) A forest plot of six genes. (D) Survival curve of CC patients in the training set. (E) Show the survival status of CC patients in the high-LMR.sig and low-LMR.sig groups and the expression of the six genes. The ROC curve depends on time for the LMR.sig in the training set (F), internal verification set (G), global set (H), and external verification set (I).

The predictive efficacy of LMRGs in CC was then assessed using an LMR.sig. Thirty-six candidate genes for constructing an LMR.sig were screened using LASSO analysis with a minimum lambda value of 0.0334. (Fig. 6A and B). To build the LMR.sig using the genes identified by LASSO and multivariate Cox analyses, *SLC10A2*, *THRSP*, *PTGIS*, *SLC25A17*, *PLAAT2* (*HRASLS2*), and *PIP4K2A* were used.

The LMR.sig per patient in the training and validation cohorts was calculated as LMR.sig = (2.365 * expression value of *SLC10A2*) + (1.276 * expression value of *THRSP*) + (0.112 * expression value of *PTGIS*) - (0.419 * expression value of *SLC25A17*) - (0.177 * expression value of *PLAAT2*) - (0.141 * expression value of *PIP4K2A*). These six genes are key genes. The hazard ratios for *SLC10A2*, *THRSP*, and *PTGIS* were more than 1, and *SLC25A17*, *PLAAT2*, and *PIP4K2A* were less than 1 (Fig. 6C).

The built LMR.sig successfully divided CC patients into high-LMR.sig and low-LMR.sig groups. Regarding overall survival, patients in the low-LMR.sig group performed better than those in the high-LMR.sig group (P = 2.1e-24, Fig. 6D). *SLC10A2*, *THRSP*, and *PTGIS* expression tended to be higher in the high-LMR.sig group, while the expression of *SLC25A17*, *PLAAT2*, and *PIP4K2A* tended to be higher in the low-LMR.sig group (Fig. 6E).

According to a time-dependent ROC analysis, the established LMR.sig displayed exact prediction capability over three years. The ROC curve's area under the curve (AUC) for 365-day, 1095-day, and 1825-day was 0.92, 0.91, and 0.94, respectively (Fig. 6F). It has a good prediction effect in the internal verification set (Fig. 6G), global set (Fig. 6H), and external verification set (Fig. 6I). According to a time-dependent ROC analysis, the established LMR.sig displayed exact prediction capability over five years.

These results indicate that the developed LMR.sig could predict the prognosis of patients with CC.

### 3.7 LMR.sig can be used as an independent prognostic indicator and development of an integrated monogram

**Fig. 7:**
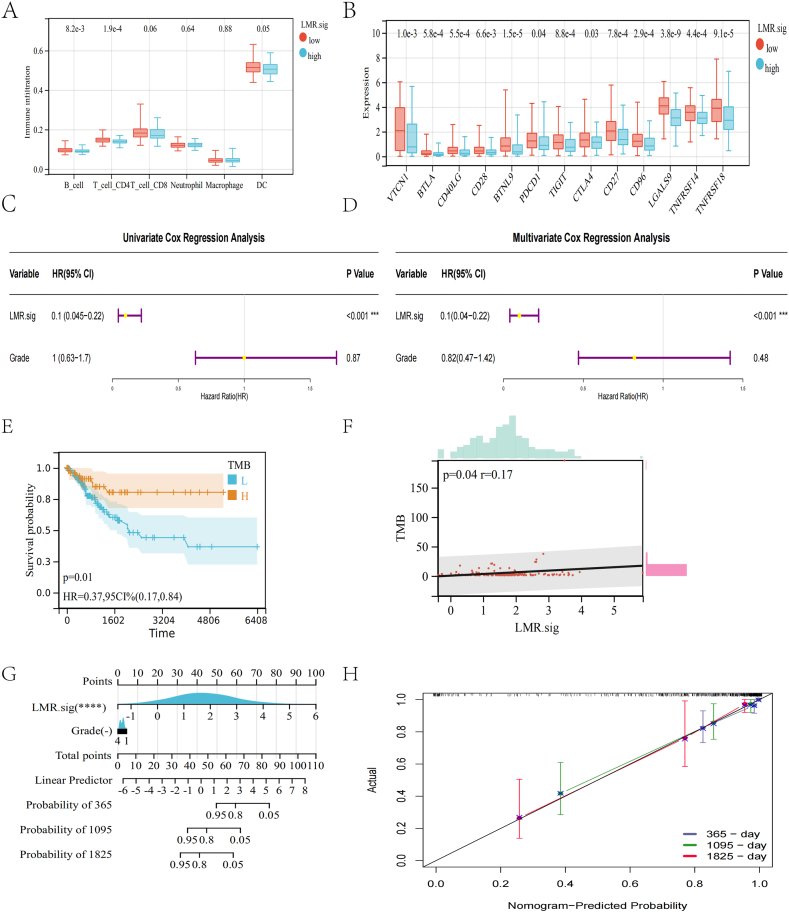
Analysis of immune infiltration, univariate and multivariate Cox analysis. (A) Analysis of immune cells in high and low-LMR.sig groups. (B) ICGs were analyzed between the two groups. Univariate (C) and multivariate (D) Cox analyses were performed for LMR.sig. (E) Analysis of KM survival in high and low TMB groups. (F) Correlation between TMB and LMR.sig. (G) Development and adjustment of a nomogram incorporating LMR.sig and clinical characteristics. (H) Adjustment of the nomogram.

We also explored the difference in immune cells between high and low-LMR.sig groups using the TIMER algorithm. Results showed that low-LMR.sig patients had significantly higher B cells (P = 8.2e-3), CD4 T cells (P = 1.9e-4), and DCs (P = 0.05) compared with high-LMR.sig patients (Fig. 7A).

We evaluated the expression of immune checkpoint genes (ICGs) in the high and low-LMR.sig groups. We found that in all the assessed ICGs, the low-LMR.sig group was significantly higher than the high-LMR.sig group (Fig. 7B).

Univariate and multivariate Cox analysis evaluated the independent prognostic value of the LMR.sig. The results showed that LMR.sig could be an independent prognostic factor in patients with CC (Fig. 7C and D).

We performed KM survival analysis by TMB of CC patients and found that the survival status of the high TMB group was significantly better than that of the low TMB group (P = 0.01, Fig. 7E). Studies have shown that higher TMB produces more neoantigens, increases T-cell recognition, and triggers anti-tumor immune responses [22]. And that cancer patients with high TMB are associated with better prognosis [23], which is consistent with our results. We also evaluated the relationship between TMB and LMR.sig in CC patients and found that TMB was significantly and positively associated with LMR.sig (P = 0.04, r = 0.17, and Fig. 7F). This further demonstrates the potential role of the LMR.sig in CC immunotherapy.

A nomogram was developed to better forecast the prognosis of CC patients. A specific score will be assigned via the created nomogram demonstrating the influence of LMR.sig and clinical factors on the prognosis of CC patients (Fig. 7G).

Regarding the model diagnostic of the nomogram, the calibration curve (Fig. 7H) suggested sufficient precision. The *C*-index for the nomogram achieved 0.8755 (95%CI: 0.8242–0.92679).

These findings demonstrated that the integrated nomogram could reliably predict the prognosis of CC patients. The built LMR.sig using LMRGs correctly predicted the prognosis of patients with CC.

### 3.8 Expression of prognostic genes and immune infiltration expression in CC

**Fig. 8:**
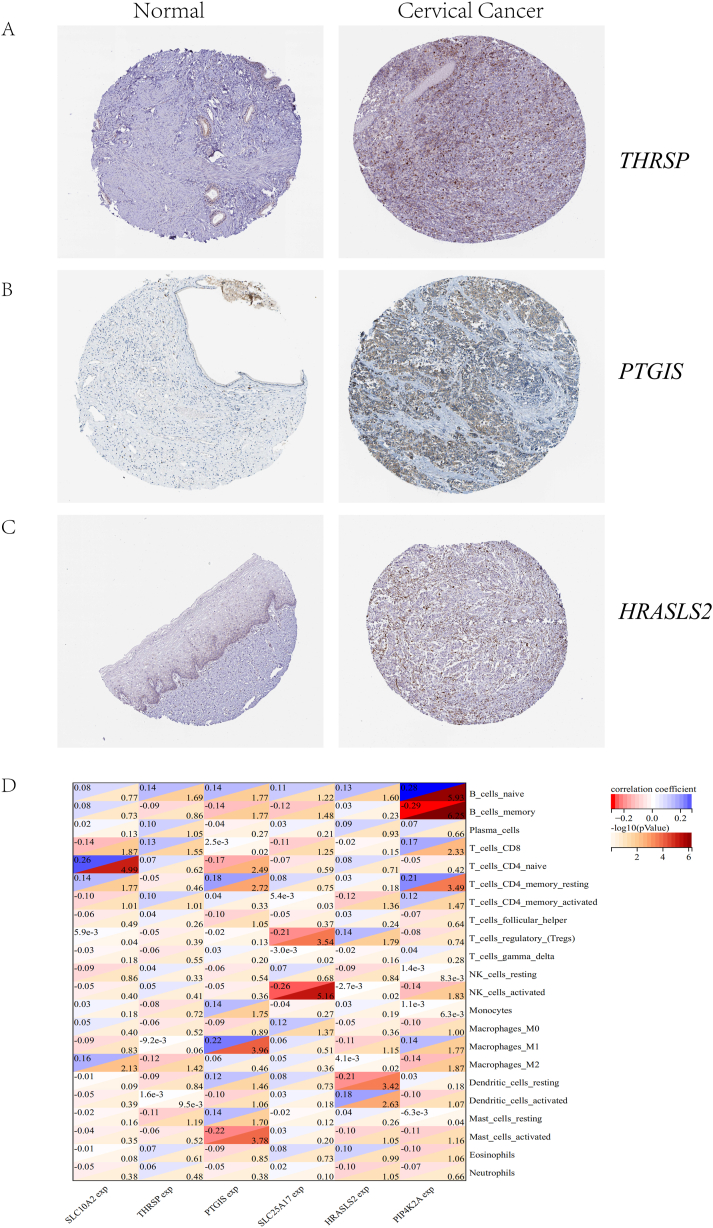
Protein expression and immune infiltration analysis of prognostic genes. Expression of *THRSP* (A), *PTGIS* (B), and *HRASLS2* (C) proteins between CC and normal tissues. (D) CIBERSORT evaluation of prognostic genes about 22 immune cells.

We evaluated the expression of *THRSP* (Fig. 8A), *PTGIS* (Fig. 8B), and *HRASLS2* (Fig. 8C) between CC and normal tissues through the HPA database. We found their protein levels were significantly higher in tumor tissues than in normal tissues.

We also evaluated the expression of six genes in immune cells in CC. We found that *SLC10A2* was significantly correlated with four types of immune cells, *THRSP* was significantly associated with three different types of immune cells, *PTGIS* was associated considerably with nine kinds of immune cells, *SLC25A17* was significantly correlated with four different types of immune cells, *HRASLS2* was significantly associated with five different types of immune cells, and *PIP4K2A* was significantly associated with eight different types of immune cells (-log10(P = 0.05) ≈1.30, -log10(P = 0.01) ≈ 2, Fig. 8D). We found that all six prognostic genes were significantly associated with different subtypes of macrophages and T cells, and *PTGIS* and *PIP4K2A* were the most widely associated with immune cells.

These results suggest that these six LMRGs are closely related to the immune infiltration of CC and can regulate the occurrence and development of CC by influencing T cells and macrophages.

### 3.9 Prognostic genes and compound docking

We used the CTD database to screen out small molecular compounds targeting prognostic genes for molecular docking studies.

Molecular docking results showed that the binding energy of Vincristine (−80.12 kcal/mol) and *SLC10A2* was the lowest, the best binding effect. *THRSP* combines best with SAYTEX 120 (−13.14 kcal/mol). While Lasiocarpine (−25.86 kcal/mol) had the best binding effect with *PTGIS, PTGIS* was also associated with T-2 Toxin (−19.10 kcal/mol) had an excellent binding effect. *SLC25A17* has the best combination effect with Adarotene (−9.15 kcal/mol). Sulforaphane (−2.84 kcal/mol) and *HRASLS2(PLAAT2)* was the best binding effect. Ivermectin (−26.27 kcal/mol) and *PIP4K2A* had the best binding effect; *PIP4K2A* was also associated with Rifampin (−25.52 kcal/mol), T-2 Toxin (−15.78 kcal/mol) and Estradiol Benzoate (−14.85 kcal/mol) all had an excellent binding effect.

**Fig. 9:**
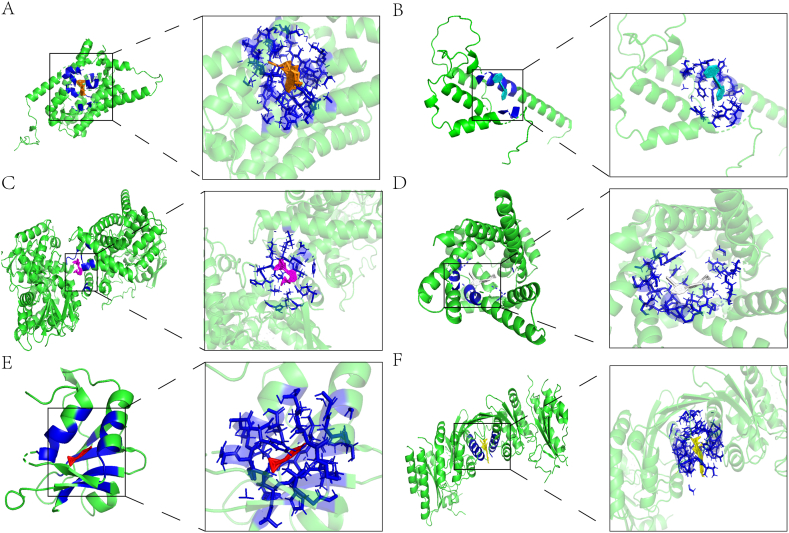
The docking results of proteins encoded by prognostic genes with small molecular compounds. (A) The docking results of *SLC10A2* with Vincristine. (B) The docking results of *THRSP* with SAYTEX 120. (C) The docking results of *PTGIS* with Lasiocarpine. (D) The docking results of *SLC25A17* with Adarotene. (E) The docking results of *HRASLS2* with Sulforaphane. (F) The docking results of *PIP4K2A* with Ivermectin.

We used Pymol to demonstrate *SLC10A2* and Vincristine (Fig. 9A), *THRSP* and SAYTEX 120 (Fig. 9B), *PTGIS* and Lasiocarpine (Fig. 9C), *SLC25A17* and Adarotene (Fig. 9D), *HRASLS2* and Sulforaphane (Fig. 9E) and *PIP4K2A* and Ivermectin (Fig. 9F) respectively.

We screened a variety of small molecular compounds that are beneficial to improve the poor prognosis caused by prognostic genes, providing a new research idea for the targeted treatment of CC.

## 4 Discussion

CC is the second most common gynecological cancer. CC, as a gynecological disease with high incidence, poses a severe threat to women's health. CC is mainly caused by HIV infection, and PIM is crucial for developing and treating CC. Metabolic changes occur extensively in cancer, and lipid metabolism is involved in cancer cell proliferation, differentiation, apoptosis, and other processes [24]. The immune microenvironment plays a significant role in the development of tumors, so it is essential to explore the immune landscape of PIM to treat CC.

We constructed the differentiation trajectories of epithelial and cancer cells using LMRGs and monocles. We found that Epithelial cells differentiated toward Cancer cells, suggesting that our lipid metabolism may be closely related to the EMT pathway.

Consensus clustering proved to be a reliable method for dividing data into different subgroups. This study identified two molecular subtypes by consistent clustering based on LMRGs. Patients in the C2 subgroup had better survival outcomes. ESTIMATE and TIMER algorithms are two effective methods to evaluate the immune landscape. These two algorithms assessed the immune landscape, and the immune status of subgroup C2 was up-regulated relative to C1. Immune infiltration assessment showed that patients with good prognoses had a relatively high immune status. This suggests that LMRGs affect patient prognosis and are closely related to the immune microenvironment. Differences in the immune landscape refer to changes in the characteristics and responses of the immune system in different individuals or disease states, and these differences can provide vital information for personalized therapeutic approaches. Analyzing patients' immune cell types, degree of infiltration, and expression levels of immune-related genes makes it possible to determine which patients may respond better to immunotherapy or other treatments (chemotherapy and targeted therapy). According to our findings stratified by the expression profile of patients' LMRGs, if the gene expression data of the tested patient is consistent with that of the C2 cluster, then it may suggest that the patient may be able to benefit from immunotherapy, which can help physicians to choose the most appropriate treatment regimen and guide the monitoring and adjustment of the treatment process.

Functional studies were undertaken to compare the two subgroups to understand the underlying biological mechanisms, and based on GO and KEGG analyses revealed that immune response regulation might modulate the role of lipid metabolism in CC development and progression. However, the precise association between lipid metabolism and immunological modulation remained unknown. As a result, we used GSEA analysis to acquire more about the underlying mechanisms. GSEA results revealed two clusters mainly enriched to alanine aspartate and glutamate metabolism. These findings suggested that the immune landscape was linked to LMRGs.

DCs are considered to be an essential mediator of anti-tumor immunity. Recent studies have also demonstrated that DC production is critical in immunotherapy response. The metabolic program of DCs determines its function and immunostimulatory capacity [25]. Fatty acid oxidation is a process used to convert fatty acids into cellular energy, which enables DCs to develop a tolerant phenotype [26,27]. These findings suggest that altered DC metabolism is essential to tumor-mediated immune escape. Macrophage activation is closely related to metabolic reprogramming. Targeting lipid metabolism in macrophages improves the prognosis of metabolic diseases. Recent evidence suggests that fatty acid oxidation is also essential for activation in M1 macrophages [28]. Our paper also found a close relationship between lipid metabolism reprogramming and DCs and macrophages.

The C2 cluster has a high survival rate and immune infiltration score. It affects the expression of some critical genes (MHC, *CD80*, *CD86*, *CD163*, *CD68,* and *CCL20*) through lipid metabolic reprogramming. It regulates the maturation and migration of immune cells (DCs and M1 macrophages) and reshapes the immunosuppressed environment of PIM. Our results were further verified by discovering that two clusters of DEGs were mainly enriched in immune function regulation pathways through functional enrichment.

In addition, we developed a predictive LMR.sig based on LMRGs and validated it in the verification cohort to accurately predict the prognosis of patients with CC. We demonstrated that LMR.sig was an independent prognostic indicator by univariate and multivariate COX analysis. A nomogram incorporating the LMR.sig and clinical variables was developed and validated, demonstrating the significant predictive ability for survival. These findings supported the importance of LMRGs in prognosticating CC. We then performed an immune infiltration analysis of these prognostic genes, respectively, further demonstrating the importance of these prognostic genes and their close association with the immune landscape.

Finally, we conducted targeted drug screening for prognostic genes. Vincristine, as a widely proven anticancer compound, has an excellent binding effect with *SLC10A2*. Vincristine has attracted much attention as a choice of adjuvant chemotherapy for CC [29]. SAYTEX 120 has good thermal stability, is commonly used as a flame retardant, and has a good combination effect with *THRSP*. Lasiocarpine is a hepatotoxic alkaloid, and its metabolic transformation intermediate, Lasiocarpine N-oxide, is a natural product with antitumor activity [30]. T-2 toxin is a potent cytotoxic toxin with antitumor properties [31]. Both compounds have an excellent binding effect with *PTGIS*. Adarotene is a synthetic retinol with proapoptotic ability and has an excellent binding effect with *SLC25A17*. Sulforaphane has been shown to have an anticancer effect on various cancers. It can synergistically enhance the activity of anticancer drugs, such as paclitaxel [32], which can become a potential anticancer drug. It has a good combination effect with *HRASLS2*. Rifampin binds well to *PIP4K2A*, which acts as a semi-synthetic antibiotic and has been shown to have antiangiogenic and antitumor effects [33]. These drugs offer a new perspective on targeted therapy through prognostic genes. Based on LMRGs, they can be studied for potential drug therapy. Our findings may aid in developing *C*C-targeted therapy and assist doctors in making more sensible treatment decisions.

Our research still needs some improvement. For starters, the conclusions of our bioinformatics research needed to be experimentally validated. Secondly, instead of utilizing our cohort, the data were gathered from several publicly accessible sources. Finally, there is a danger of bias owing to imbalanced clinicopathological features and treatment heterogeneity since all of the participants in this research were selected retrospectively. More prospective studies are required to validate the prognostic usefulness of LMRGs in CC.

## 5 Conclusion

We reveal lipid metabolism scores in CC single-cell landscapes. We also found that lipid metabolism may be closely related to EMT. Using consensus clustering, two molecular subtypes of CC were identified. According to immunological and functional assessments, LMRGs affect the immune landscape of PIM. We found that lipid metabolic reprogramming can improve the expression, maturation, and migration of immune cells, reshape the immunosuppressive environment of PIM in CC patients, and improve the survival time of patients. We construct an LMR.sig based on LMRGs. Several drugs targeting prognostic genes have been found. Some contributions have been made to discovering biomarkers for CC, which can help develop new targeted drugs and risk stratification for CC patients.

## Availability of data

The data sets used in this study are all available in online public databases.

## Consent for publication

Not applicable.

## Ethics approval and consent to participate

Not applicable.

## Funding

1. Special fund project for central government leading local science and technology development（202207AB110015).
2. The medical edible flower innovation team of Yunnan colleges and universities (project No. 2020YGC01).
3. 10.13039/501100007301Kunming University of Science and Technology Medical joint project (grant number KUST-PE2022005Y).

## CRediT authorship contribution statement

**Yongzhi Chen:** Conceptualization. **Rongjie Cui:** Conceptualization. **Dun Xiong:** Funding acquisition. **Yuan Zhao:** Funding acquisition. **Jianyu Pang:** Methodology. **Samina Gul:** Software. **Qi Qi:** Software. **Yuheng Tang:** Software. **Xuhong Zhou:** Funding acquisition. **Wenru Tang:** Conceptualization.

## Declaration of competing interest

The authors declare that they have no known competing financial interests or personal relationships that could have appeared to influence the work reported in this paper.

## Figures and Tables

**figs1:**
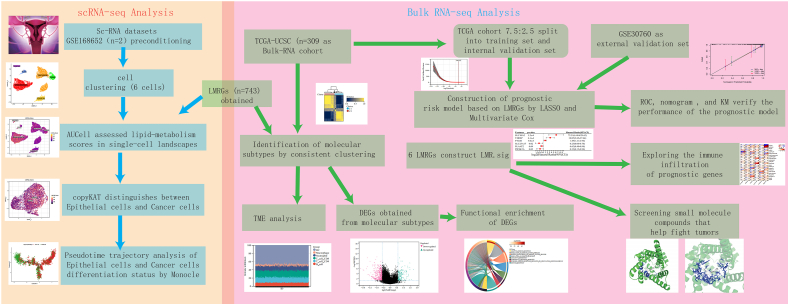


**figs2:**
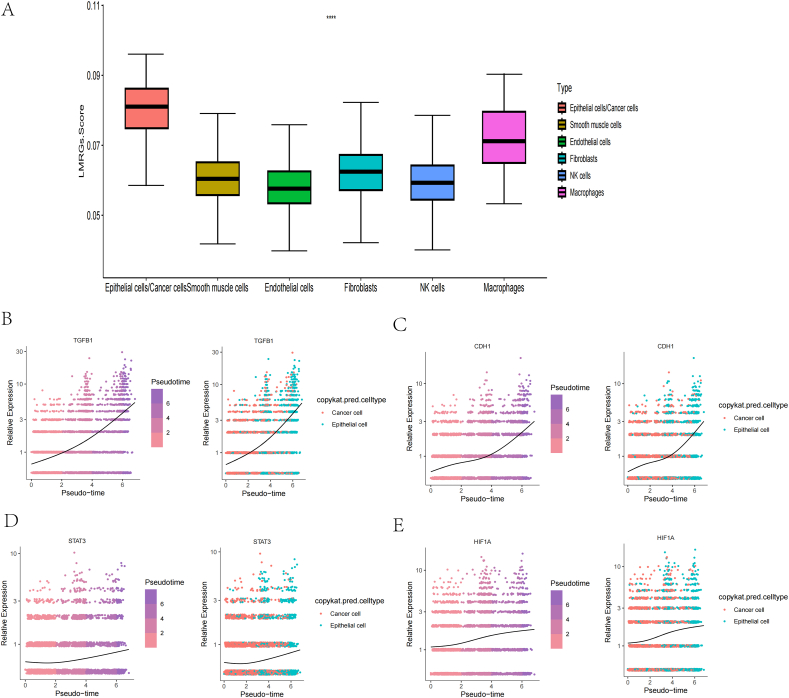


**figs3:**
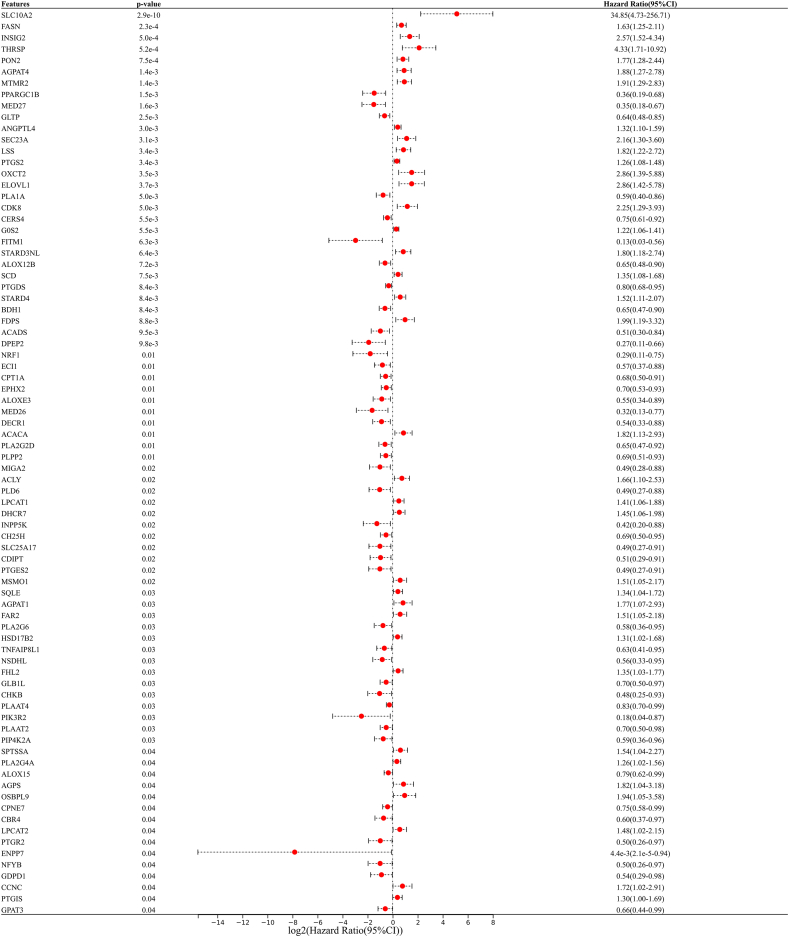


**figs4:**